# Advanced Complex Analysis of the Thermal Softening of Nitrided Layers in Tools during Hot Die Forging

**DOI:** 10.3390/ma14020355

**Published:** 2021-01-13

**Authors:** Jakub Krawczyk, Paweł Widomski, Marcin Kaszuba

**Affiliations:** Department of Metal Forming, Welding and Metrology, Faculty of Mechanical Engineering, Wroclaw University of Science and Technology, Lukasiewicz Street 5, 50-371 Wroclaw, Poland; jakub.krawczyk@pwr.edu.pl (J.K.); marcin.kaszuba@pwr.edu.pl (M.K.)

**Keywords:** thermal softening, nitrided layer, hot forging

## Abstract

This article is devoted to the issues of thermal softening of materials in the surface layer of forging tools. The research covers numerical modeling of the forging process, laboratory tests of tempering of nitrided layers, and the analysis of tempering of the surface layer of tools in the actual forging process. Numerical modeling was supported by measuring the temperature inside the tools with a thermocouple inserted into the tool to measure the temperature as close to the surface as possible. The modeling results confirmed the possibility of tempering the die material. The results of laboratory tests made it possible to determine the influence of temperature on tempering at different surface layer depths. Numerical analysis and measurement of surface layer microhardness of tools revealed the destructive effect of temperature during forging on the tempering of the nitrided layer and on the material layers located deeper below the nitrided layer. The results have shown that in the hot forging processes carried out in accordance with the adopted technology, the surface layer of working tools is overheated locally to a temperature above 600 °C and tempering occurs. Moreover, overheating effects are visible, because the surface layer is tempered to a depth of 0.3 mm. Finally, such tempering processes lead to a decrease in the die hardness, which causes accelerated wear because of the abrasion and plastic deformation. The nitriding does not protect against the tempering phenomenon, but only delays the material softening process, because tempering occurs in the nitrided layer and in the layers deeper under the nitrided layer. Below the nitrided layer, tempering occurs relatively quickly and a soft layer is formed with a hardness below 400 HV.

## 1. Introduction

Tools in the die forging processes are subjected to many destructive factors that contribute to their premature wear. The reasons can be found in the extremely difficult working conditions of the tools, which are subjected to cyclically changing high temperatures and pressures [1]. The pressures are accompanied by the movement of the forging material on the tool surface. This creates a friction pair in which the surface of tools is subject to abrasive wear, which is further intensified by hard oxide particles flaking off the surface of hot charge material [2]. Forging tools work at high temperatures, which are also cyclically variable, the surface tool layer is subjected to alternating compressive and tensile stresses while cooling the cavity. As a result, structural changes occur in the material. Initially, there is a temporary strengthening of the tool material during plastic deformation of the die in subsequent cycles [3]. This strengthening decays as a result of thermally activated processes causing progressive coagulation of carbides and die recrystallization. This leads to the phenomenon of heat checking and thermo-mechanical fatigue, which causes cracking on the die surface [4]. In the cracked zone, accelerated oxidation takes place, crack propagates deeper from the surface as well as its widening, which over time contributes to the formation of the first abrasive grooves. Immense amounts of heat transferred from the forging to the tools can also lead to overheating of the tool surface layer. This is the cause of tempering and, consequently, loss of hardness of the tool material, where one of the basic features that forging tools should have for hot forging is high hardness while maintaining sufficient crack resistance [5,6].

Therefore, by definition, forging tools should be resistant to the tempering effects of high temperatures that occur during forging. The tempering mechanism is dependent on the diffusion of carbon and alloying elements and is closely related to temperature and time. The influence of temperature and tempering time on steel hardness is presented by the tempering parameter M—J.H. Hollomon and L.D. Jaffe [7]:

M = T(C + log t)(1)

where:

T—tempering temperature on the absolute scale, K,

t—tempering time in seconds,

C—constant depending on carbon concentration.

Usually, the notion of temperability is limited to expressing the dependence of hardness on the tempering temperature at a constant time of this operation, and such relationships are determined under laboratory conditions. Graphs showing the dependence of hardness on the tempering temperature for individual tool steel grades are published in the technical literature and material data sheets [8]. In actuality, however, often the operating temperature range of the tool exceeds the tempering temperature range of the tool material. This may cause physical and structural changes in the material and thus disqualify the tool from further use. Moreover, exposure to high temperature is accompanied by high pressures, which may additionally decrease the hardness due to tempering [9].

One of the basic measures aimed at improving the performance of the surface layer of the parts, including forging tools, is nitriding. By increasing the surface layer hardness, nitriding increases the tool resistance to abrasion, fatigue strength, and corrosion resistance. Observations of many industrial forging processes in which nitrided tools were applied have proven that this treatment makes it possible to increase the tool service life several times [10,11]. Nevertheless, nitriding by itself does not protect the material from the adverse effects of high temperature. Attempts made for this purpose concerned the use of hybrid layers such as a nitrided layer/PVD coating, where the purpose of the coating, apart from increasing resistance to abrasive wear, is also insulation protecting the material against tempering under the influence of high temperature. Because of the very small thickness, the insulating effect is very short and, moreover, when the PVD coating is damaged, the tool material is no longer protected [12].

Another method of increasing the tool resistance to exposure of high temperature is the use of hard-faced layers, where the use of a hard-faced layer resistant to tempering in the temperature range of the tool with a relatively large thickness effectively insulates the basic metal [13].

Special lubricant/coolants are also used, which reduce the friction between the tool and the forged part as well as cool the tool. In addition, after application, these agents create a lubricating film that effectively reduces the amount of heat that enters the tool from the hot workpiece material. For steel forging graphite-based lubricant/coolants are most often used, the heat transfer coefficient values of individual media vary depending on the type of graphite used [14].

Despite the efforts to reduce the effect of heat on the tool surface, tempering of the forging tool surface layer is commonly observed in forging processes [15]. This article deals with the research on the phenomenon of tempering of nitrided layers, as the most commonly used to increase the durability of forging tools.

## 2. Materials and Methods

The experiment first consisted in analyzing the temperature of forging tools operated in the hot forging process. For this purpose, measurements were made with a thermocouple and a thermal imaging camera. Then, a numerical model was built to determine the maximum values of temperature in function of time on the surface and in the surface layer which was impossible to be measured. The next stage was the laboratory tests of the influence of temperature and tempering time on the hardness of nitrided layers, which are usually used on forging tools. The last stage was the study of forging dies that worked for a specified number of forging cycles. The hardness and microstructure of the dies were tested, and then the temperature and time at which the tools worked were assessed. The results were compared to those obtained by annealing in laboratory conditions.

### 2.1. Testing Tempering of Nitrided Layers under Laboratory Conditions

The tests were carried out on samples made of X37CrMoV5-1 hot work tool steel, subjected to quenching and tempering, the same as in the case of forging tools consisting in hardening and double tempering at 600 °C. The hardness after heat treatment was 43–45 HRC. Then the samples were gas nitrided. To reproduce the effect of the temperature that occurs in the actual process on the nitrided layer, the treated samples were annealed at the temperature of 500, 550, 575, 600, 625, 650, 675, and 700 °C [16]. The adopted annealing time was 2 h and 4 h. During forging, the tool remains in contact with the hot material for a period of approximately. 1 to 1.3 s, which means that for 10,000 forging cycles it remains in contact with the hot forging for a period of time of up to 4 h. After sample annealing, the hardness distribution as a function of the distance from the surface was determined. The hardness was measured using LECO LM100AT (St. Joseph, MI, USA) microhardness tester. The measurement was performed using the Vickers method with a load of 100 g. The purpose of these tests was to experimentally demonstrate the effect of specific temperatures on the hardness of nitrided layers and the die core material. The test result was the development of tempering curves of the surface layer at different depths. The obtained curves can be useful in numerical modeling of the nitrided layer in the aspect of modeling forging tool wear.

### 2.2. Analysis of Tool Working Conditions in a Selected Industrial Forging Process

The analyzed forging process is carried out on a Massey 2500T (Massey Forging Ltd., Manchester, UK) press with the speed of 60 strokes per minute in three operations. It is a Maxi-type mechanical crank press that is most popular in hot forging processes and has been chosen as a representative example of a forging press. All tools are preheated to a temperature of approximately. 250 °C by prolonged contact with preheated preforms.

The dies are made of X37CrMoV5-1 hot work tool steel, quenched and tempered (quenching and double tempering at 600 °C) to a hardness of 43 to 45 HRC and gas nitrided.

Each of the three operations involves top and bottom tools, each of which has slightly different durability. The preliminary forging tools (2nd operation) are characterized by the lowest durability, usually they are destroyed on average after approximately 7000 to 8000 cycles, according to the data obtained from the production plant (Kuźnia Jawor) [17].

**Figure 1:**
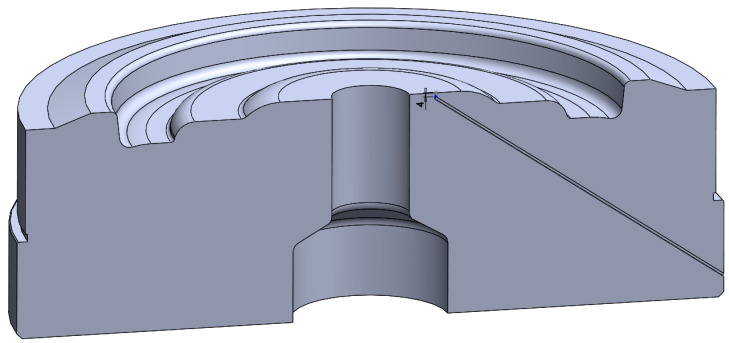
Method of temperature measurement in the tool during die forging.

The key parameter affecting the tempering of dies is their working temperature. In order to determine the actual temperature prevailing on the surface and in the surface layer of the tools, the temperature was measured using a thermal imaging camera (before and after the forging process) and measured with a thermocouple inserted through an additional hole in the tool, approximately 4 mm below the surface. The choice of the measurement location is due to the fact that it is the most heated area of the forging die. The distance of 4 mm from the surface is due to the necessity of not to damage or weaken the surface, which works under high-stress conditions. The thermocouples T-208p-K-2-2000-40-3-SP-1-3-S-1150 °C were used to measure the temperature in the data recording system with a frequency of 100Hz. The measurement with the FLIR thermal imaging camera was performed with the emissivity parameter of 0.95. Figure 1 shows schematically the measurement system with the place of measurement marked.

As the temperature measurement with a thermal imaging camera is not possible at the moment of contact with the forging, when the temperature on the tool surface is the highest, numerical modeling was proposed to determine this value. Similarly, measuring with a thermocouple makes it possible to measure the temperature at a certain depth below the surface which is a valuable piece of information but does not give the answer to what the maximum temperature in the surface layer is. Nevertheless, the data from both measurements served as boundary values for building and adjusting the numerical model so that the model was as close as possible to actual conditions. The obtained temperature measurement results can be used as boundary values for the developed model [18].

**Figure 2:**
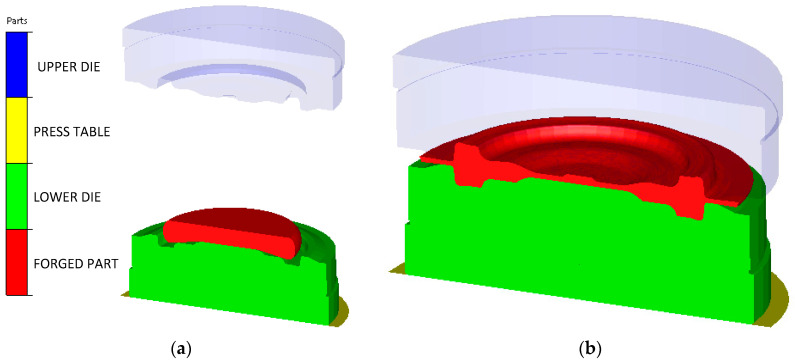
General view of the FEM model: (**a**) before; (**b**) after 2nd forging operation.

The axially symmetric numerical model of the successive cycles of two forging operations was developed using the Forge NxT 3.0 program. In the simulation, the forging and the bottom die insert were developed as deformable bodies. The top die and the bottom table were created as a non-deformable body in order to reduce the computation time and because this study deals with the temperatures on the bottom die. The general view of the model before and after the 2nd forming operation is shown in Figure 2.

Based on the observation of the actual process, the single forging cycle was divided into stages and the average times of each stage were determined. The forging time of one forging (3 operations) takes approximately 14 s. The forging cycle in the second operation consists of the following stages:

1. Contact with the bottom die—0.37 s;
2. Deformation—0.06 s;
3. Contact with the bottom die before transfer—0.58 s;
4. Free self-cooling—2 s;
5. Lubrication—0.7 s;
6. Free self-cooling for the next cycle—10.29 s.

Based on the temperature measurement in the actual process, the following boundary conditions were adopted for the model development:

- The initial charge temperature is the same as after the forging was transferred from the 1st to the 2nd forging operation—it was exported from the simulation of the first operation (upsetting);
- Die initial temperatures—bottom—250 °C, upper—350 °C (its temperature does not change during calculations);
- Heat transfer coefficients in contact—14 kW/m^2^ × K between the dies and the forging;
- Heat transfer coefficient of the bottom die working surface with the environment during lubrication/cooling 2.6 kW/m^2^ × K, during free self-cooling 15.5 W/m^2^ × K;
- Friction coefficient—Coulomb model marked in the program as water + graphite—µ = 0.15.

### 2.3. Analysis of the Influence of Working Temperature on the Phenomenon of Tool Thermal Softening in a Selected Industrial Forging Process

The influence of the working temperature on the tool thermal softening phenomenon was tested on tools that had worked a different number of forging cycles in the range from 1500 to 8000. First, the hardness distributions of the surface layer as a function of the distance from the surface were determined. Hardness measurements were carried out in the same place where the temperature during forging was measured and determined in modeling. Then, based on the results obtained, the surface layer was divided into four characteristic zones—layers at a depth of 0.05 mm, 0.1 mm, 0.15 mm, and 0.2 mm. The next step was to assign the maximum temperatures that occur in individual zones and exposure times to temperatures above 600 °C. The determined values allowed for the analysis of the impact of the holding time and temperature on the change in hardness and structural changes in the nitrided layer and in the layers directly under the nitrided layer. Examinations of the microstructure, were done using a Keyence VHX-6000 digital microscope (Osaka, Japan) with magnification up to 1000×, with the capability of measurement under a variable lighting angle, depth of field composition in 2D and 3D, HDR plus technology, in which photographs of the microstructure were taken.

## 3. Results and Discussion

The first part presents the results of the tempering of nitrided layers under laboratory conditions. Then, the results of a comprehensive analysis of the actual working temperature of tools in the forging process are included. This analysis was supported by numerical modeling of the forging process. The effect of the tests are the measuring results of microhardness and microstructure in actual tools used in the forging process. These tests determined the effect of heat on the tempering of individual material layers in the nitrided surface layer of forging tools. Recent studies confirm the significance of tempering effect on the wear of hot forging tools [19]. Therefore, the following results are up-to-date and expected in the scientific community.

### 3.1. Test Results for Tempering of Nitrided Layers under Laboratory Conditions

**Figure 3:**
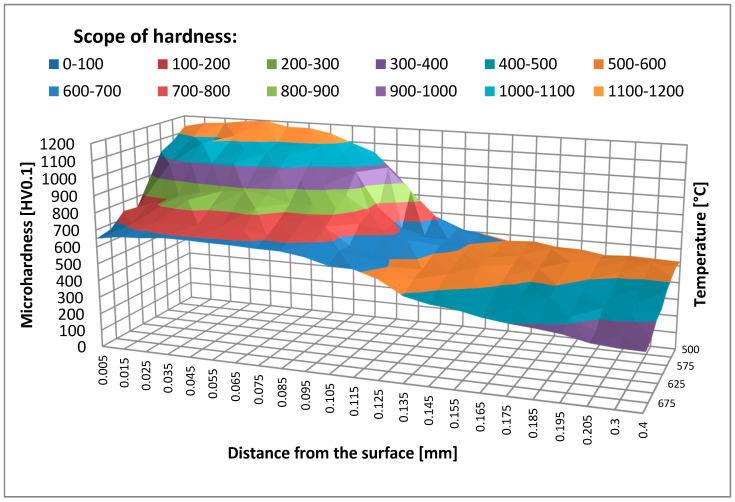
Hardness in the surface layer of samples after annealing for 2 h as a function of the distance from the surface.

**Figure 4:**
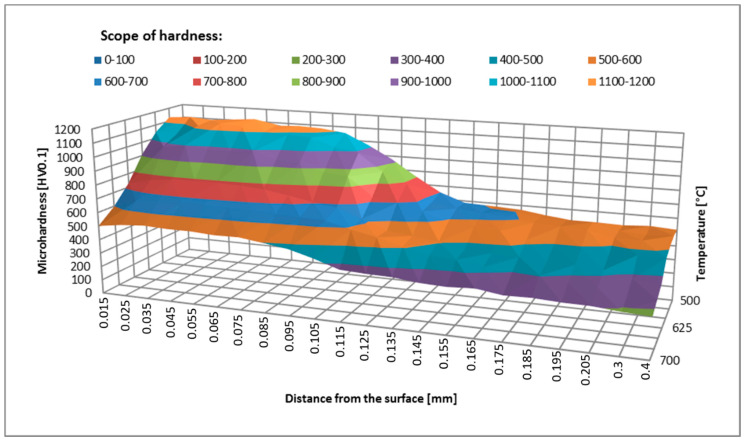
Hardness in the surface layer of samples after annealing for 4 h as a function of the distance from the surface.

The samples were held at temperatures ranging from 500–700 °C for 2 and 4 h. After annealing, the hardness in the near-surface area was measured. The hardness measurement results for samples held at different temperatures are shown in Figure 3 and Figure 4.

The hardness measurement results show a distinct tempering effect in the nitrided layer and below the hardened layer in the core material. As temperature increases, hardness decreases, the greatest decrease being recorded at temperatures above 600 °C. The temperature of 600 °C can be considered critical (the one that must not be exceeded), which is consistent with the data contained in the literature [20] and with the guidelines of tool steel manufacturers [21]. Changes in the material hardness are also related to the holding time. By comparing the graphs in Figure 3 and Figure 4, a conclusion may be drawn that hardness depends on temperature and time. The results of nitrided layers tempered under laboratory conditions can be used as a reference in the evaluation of the tempering process taking place in the actual forging processes. Based on these results it is possible to approximate at what temperature and for what period of time a forging tool has worked. This comparison can be distorted in the layers closest to the surface because in this zone the tempering is affected by the high pressure exerted and a number of physicochemical factors that do not exist under laboratory conditions during annealing [22].

### 3.2. Results of Temperature Analysis of Tools in the Die Forging Process

**Figure 5:**
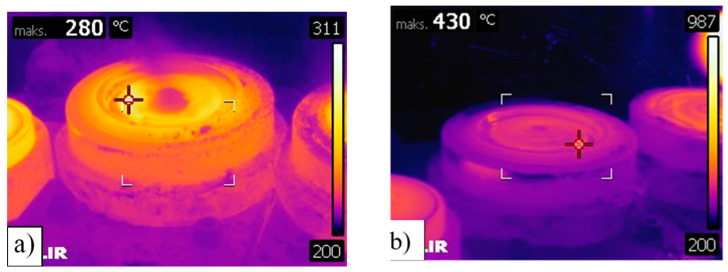
Thermogram of the die working surface: (**a**) Just before forging, after lubrication and cooling; (**b**) immediately after removing the forging from the die surface.

In order to determine the value of the tool working temperature, the measurement data were used first. Temperature measurement results include photos from a thermal imaging camera and data from the measurement using the thermocouple. Figure 5 shows the photos from a thermal imaging camera measured on the die working surface just before forging and immediately after removing the forging from the die surface.

**Figure 6:**
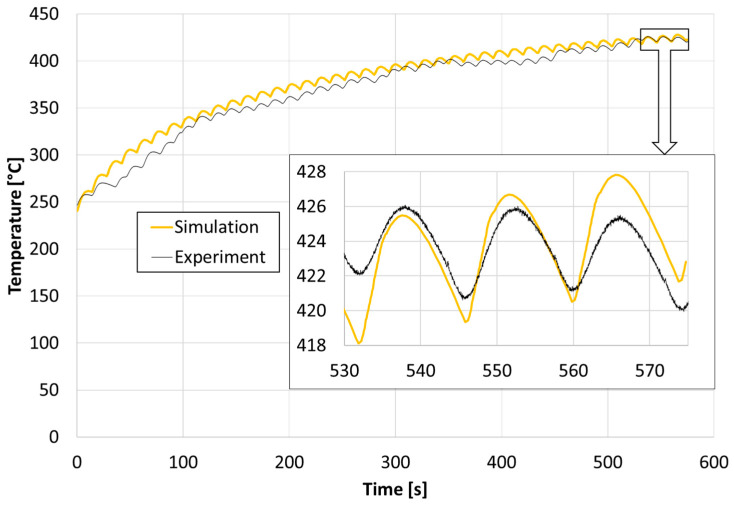
Comparison of temperature measured by thermocouple at a depth of 4 mm below the surface and from a sensor placed in the same place in the simulation.

The results of the thermal imaging measurement indicate the presence of very high temperature amplitudes on the surface (over 150 °C difference during one forging cycle). These differences result mainly from heating during contact with the forging and intensive cooling by spraying aqueous graphite suspension. During forging, the temperature may be much higher, which is also indicated by the results of the temperature measurement in the tool, measured with a thermocouple inserted into the tool, in accordance with the methodology described in Section 2. The measurement results for a single forging cycle, measured approximately 4 mm below the surface, are shown in Figure 6, for the period of the first 10 min of tool operation after pre-heating to 250 °C.

The results of the tool temperature measurement at a depth of 4 mm in a single forging cycle show variation in the range of 420 to 426 °C. These are very small changes compared to the changes in the range of 280 to 430 °C observed on the surface in the measurement using a thermal imaging camera. The results presented in Figure 6 demonstrate that the tool-operating temperature stabilized after approximately 41 forging cycles. Because of the nature of the hand forging process, the temperature cycles are irregular.

The performed measurements made it possible to determine the temperature on the die surface, but not during contact with the hot forging (only before and after forging). Also, the measurement using a thermocouple only showed a temperature at 4 mm below the surface. These results are valuable, but numerical modeling was used to determine the maximum temperatures in the tool surface layer during forging. The obtained temperature measurement results were used to determine the boundary conditions for the developed model.

In order to compare the temperature measured with a thermocouple at a depth of 4 mm from the tool working surface with the results of numerical computation, a sensor was indicated in the model that reads the temperature from a selected place in the numerical simulation—its location corresponded to the position of the thermocouple measuring tip in actual tests. Figure 6 shows a comparison of the temperature curves from the actual forging process with the simulation results.

Having analyzed the graphs of Figure 6, it can be noticed that these waveforms correlate well with each other, after stabilizing the temperature during tests and simulations—after about 41 forging cycles.

**Figure 7:**
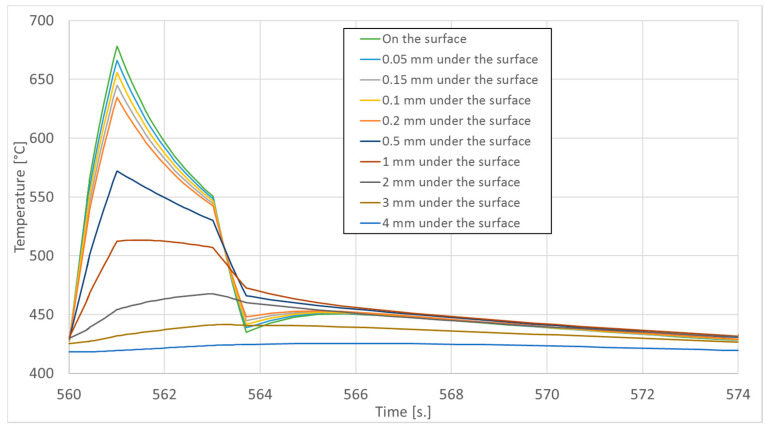
Temperature graph in the last (41st) cycle in the die in the second forging operation.

In order to determine the temperature on the surface and at the depths of 0.05 mm, 0.1 mm, 0.15 mm, 0.2 mm, 0.5 mm, 1 mm, 2 mm, 3 mm, and 4 mm sensors were placed there in the numerical model. The temperature graphs in the last (41st) forging cycle in these areas are shown in Figure 7.

As shown by the temperature waveforms in Figure 7, during forging, the temperature rises locally on the surface from approximately 430 °C to approximately 680 °C. So high temperature can lead to tempering of the surface layer of the forging tools.

**Figure 8:**
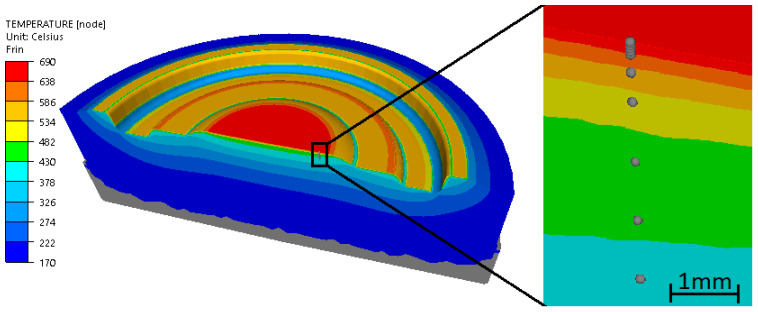
Temperature distribution in the last (41st) of two forging operations—the last forming step and the location of sensors in the numerical model.

The temperature distribution after 41 forming cycles in the last forging step on the bottom die insert is shown in Figure 8. The figure also shows the location of the sensors from which the temperature was read in the numerical simulation.

As can be seen in Figure 8, the highest temperature occurs in the central part of the bottom die under consideration and this is where die tempering is expected.

### 3.3. Test Results for Tempering of Nitrided Layers under the Conditions of the Actual Forging Process

**Figure 9:**
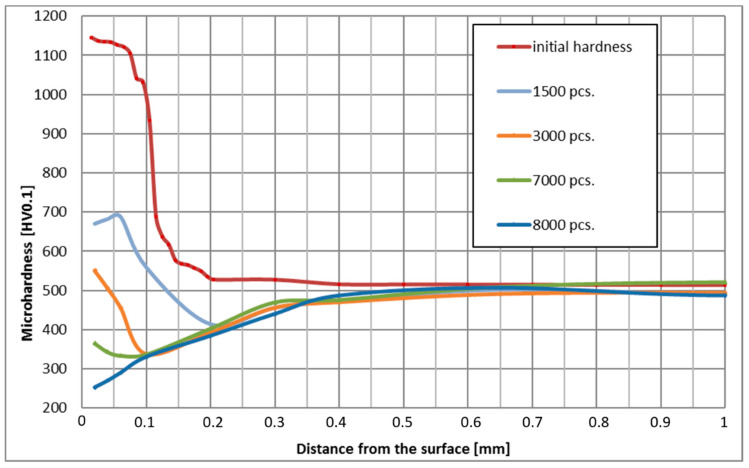
Results of microhardness measurement of nitrided surface layers after forging.

The influence of temperature and tool holding time on tempering was tested for four representative forging tools which have worked 1500, 3000, 7000, and 8000 forging cycles. The number of cycles was chosen as it was considered “representative,” based on experience. The number of forging cycles results from conditions of production process and numbers of ordered forgings. Figure 9 shows the result of the microhardness measurement in the surface layer of these tools, measured perpendicularly to the surface, according to the vector consisting of none measuring points located at 0.02, 0.04, 0.06, 0.1, 0.2, 0.3, 0.4, 0.65, and 1 mm of distance from the surface, for which the tool working temperature was also determined.

The results shown in Figure 9 confirm the effect of heat at depths of up to 0.5 mm. This depth is much greater than the effective thickness of the nitrided layer obtained with the commonly used regular gas nitriding technology, which is usually at the level of 0.15 mm. These results reveal premature (after 1500 forgings) tempering of the nitrided layer and of the layers of material below the nitrided layer. Based on these data, four characteristic zones were determined, in which a detailed analysis of temperature changes over time as well as changes in the microstructure and properties of the surface layer were carried out. The designated zones are layers of material at a depth of 0.05 mm, 0.1 mm, 0.15 mm, and 0.2 mm.

In each of the zones, the maximum operating temperature and the of exposure time to the temperature above the maximum operating temperature of the specification, i.e., above 600 °C, were determined based on the temperature analysis presented in Figure 7. These results are summarized in Table 1.

The analysis of the influence of both of the aforementioned factors on the tempering of forging dies was carried out in the four mentioned zones. For each zone, a graph of temperature versus time, initial hardness, and hardness after 1500, 3000, 7000, and 8000 cycles was determined. Moreover, an examination of the microstructure was made to verify changes in the microstructure of the surface layer.

The first zone is a layer approximately 0.05 mm below the surface. There, hardness of approximately 1140 HV was obtained by nitriding. The maximum working temperature in this place was approximately 666.1 °C and the holding time at a temperature above 600 °C was 30 to 157 min. The results are shown in Table 2.

Based on the data obtained, the effect of the maximum operating temperature is much more significant than the effect of the holding time at temperatures above 600 °C. The layer operating at a temperature of 666.1 °C was ultimately softened significantly below 300 HV. The hardness of the layers that were not held so intensely decreased to 330 HV at 655 °C, 357 HV at 645 °C, and 384 HV at 635 °C (Table 2). The hardness reduction phenomenon was gradual and dependent on the holding time, as evidenced by the results presented in Table 1. Holding for 2 h under laboratory conditions did not lead to such a radical decrease in temperature, while after 4 h both at a depth of 0.05 mm and 0.1 mm, a decrease in hardness to 600–700 HV was observed (Figure 4). The reason for such a discrepancy may be the operating temperature in the forging process being temporary slightly exceeded due to cooling errors or difficulties in removing the forging from the die cavity. Then, in the case of such a high operating temperature, additional unexpected die heating can easily lead to overheating even above 700 °C.

**Figure 10:**
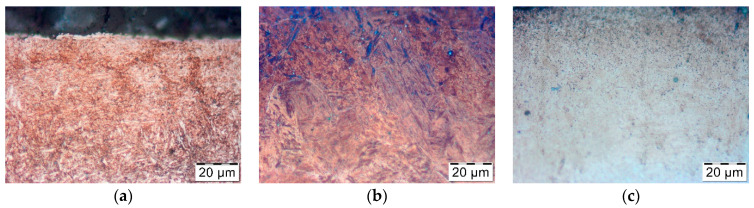
The microstructure of the nitrided layer: (**a**) as delivered; (**b**) held at 675 °C for 2 h; (**c**) after 3000 forging cycles at approximately 666.1 °C.

The thermal softening process in nitrided layers at a depth of 0.05 mm and 0.1 mm at a temperature of approximately 666.1 °C led to a hardness decrease below the limit value of 500 HV only between 1500th and 3000th forging cycle, which corresponds to the holding time of approximately 1 h. Under laboratory conditions, such intensive tempering took place at the temperature of 700 °C (Figure 3). Changes in the microstructure during tempering were also analyzed. Figure 10 below shows the microstructure of the nitrided layer as delivered and tempered at 675 °C for 2 h, and the nitrided layer in the tool after 3000 forging cycles at approximately 666.1 °C.

The tempering phenomenon leads to the coagulation of cementite and other carbide precipitates, which can be observed in the nitrided layer in the form of small black dots. In the case of the die-derived sample (Figure 10c), coagulated carbide precipitates are more numerous at the near-surface layer where annealing at higher temperature occurs. In the case of a sample annealed entirely in laboratory conditions, these changes are visible in its entire volume. The recent research results confirm such a negative effect of annealing of nitride layers on X37CrMoV51 tool steel [23].

**Figure 11:**
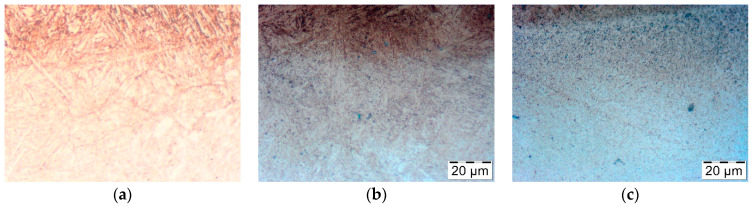
The microstructure at a depth of approximately 0.2 mm (below the nitrided layer) (**a**) as delivered, (**b**) held at 625 °C for 2 h (**c**) after 3000 forging cycles at approximately 625 °C.

On the other hand, in the layers lying at a depth of 0.15 mm and below, where the hardness was initially approximately 550 HV, overheating led to tempering and a reduction in hardness of 350 to 400 HV. The reason for this change is undoubtedly holding at a temperature in the range of 600 to 645 °C for 30 to 120 min. Also, annealing under laboratory conditions at similar temperatures for 2 h at a similar depth led to a reduction of hardness to 350 to 450 HV (Figure 3). Similar to the nitrided layers, the microstructure was also analyzed in this zone. Figure 11 below shows the view of the microstructure of the layer at a depth of approximately 0.2 mm (below the nitrided layer) as delivered and tempered at 625 °C for 2 h, and the same layer of material from a die that worked 3000 forging cycles at approximately 625 °C.

In the microstructure of the non-annealed nitrided layer, precipitates of cementite and other carbides in the material are sparse and dispersed (Figure 11a). In contrast, in the case of the laboratory annealed sample (Figure 11b), numerous coagulated precipitates appear, which are located throughout the entire volume of the material. In the sample coming from the forging die, these precipitates are mainly located directly under the nitrided layer, which proves that these parts of the material are overheated. This is confirmed by the results of temperature measurement and hardness measurement, which show that the material is thermally softened during forging at a depth of up to 0.5 mm below the surface.

## 4. Conclusions

The research on the thermal softening process of the surface layer of nitrided dies during hot forging lead to the following conclusions.

The tests conducted have shown that in the hot forging processes carried out in accordance with the adopted technology, the surface layer of working tools is overheated locally. The material in the surface layer on the surface and directly below the surface is temporarily heated to a temperature above 600 °C and tempering occurs. Overheating occurs in the short time during contact with the forging and therefore cannot be observable. However, overheating effects are visible, because the surface layer is tempered to a depth of 0.3 mm. Consequently, tempering leads to a decrease in the die hardness, which causes accelerated wear due to abrasion and plastic deformation [8].

The working temperature and the holding time have a paramount effect on the tempering phenomenon. The critical parameter in this case is exposure to high temperatures above 600 °C. It was observed that even if the exposure time is very short, even short thermal cycles significantly decrease hardness when the material temperature exceeds 600 °C. For higher temperatures in the range of 650 to 700 °C, diffusion phenomena occur much faster and steel hardness decreases even below 300 HV.

The nitriding does not protect against the tempering phenomenon, but only delays the material softening process. Furthermore, during forging tempering occurs in the nitrided layer and in the layers deeper under the nitrided layer. Below the nitrided layer, tempering occurs relatively quickly and a soft layer is formed with a hardness below 400 HV. This may cause plastic deformation of the entire surface layer and cracking of nitrided layers due to the loss of base metal stability [24].

One should be aware of the real risk of overheating and using more effective cooling, as well as reduce the time of contact with the hot material of forgings. Moreover, it is recommended that the average temperature observed on the die surface should not exceed 400 °C (excluding the moment of contact with the forging). Then the risk of reaching critical forging values exceeding 600 °C is reduced. Otherwise, for the observed temperatures above 400 °C in the time without contact with the forging, during the contact, as in the analyzed case, there will be temporary overheating up to 700 °C.

In forging processes carried out in short (sometimes automatic) cycles, tool materials or hard-faced layers with greater resistance to high temperature should be used. A thin layer with a minimum thickness of 1 mm as more resistant to tempering should suffice, because overheating in this case concerns only the surface layer at a depth of 0.5 mm.

## Figures and Tables

**Table 1:** Working conditions of the forging die at different depths.

Depth (mm)	Max. Working Temperature (°C)	Tempering Time at Temperature above 600 °C (min)				
		in 1 Cycle	in 1500 Forging Cycles	in 3000 Forging Cycles	in 7000 Forging Cycles	in 8000 Forging Cycles
0	678.1	1.31 s	33	66	153	175
0.05	666.1	1.18 s	30	59	138	157
0.1	655.6	1.01 s	25	51	118	135
0.15	645.1	0.90 s	23	45	105	120
0.2	634.7	0.71 s	18	36	83	95

**Table 2:** Hardness of dies at indicated analyzed points.

Depth (mm)	Max. Working Temperature (°C)	Hardness (HV)				
		Initial	after 1500 Forging Cycles	after 3000 Forging Cycles	after 7000 Forging Cycles	after 8000 Forging Cycles
0.05	666.1	1130	685	481	338	279
0.1	655.6	981	559	337	337	330
0.15	645.1	572	476	365	370	357
0.2	634.7	530	414	392	403	384
